# Modeling of Photochemical Reactions in a Focused Laser Beam

**DOI:** 10.6028/jres.112.016

**Published:** 2007-08-01

**Authors:** A. K. Gaigalas, F. Y. Hunt, L. Wang

**Affiliations:** National Institute of Standards and Technology, Gaithersburg, MD 20899

**Keywords:** flowing solution, frequency domain, mathematical modeling, modulated laser illumination, photodegradation, singular perturbation technique

## Abstract

Fluorescent materials play a prominent role in the qualitative and quantitative measurement of scientific phenomena of importance in biotechnology and biomedical applications. Photodegradation of fluorophores is a process that determines the accuracy and sensitivity of such measurements. This is the motivation for developing methods for accurately measuring fluorophore photodegradation rates. Recently, illumination consisting of short pulses has been used to examine the decay of photochemical reaction products. However, the time resolved measurements are difficult to interpret since the photodegradation process usually involves multiple time scales. The frequency domain measurement technique discussed here looks at the frequency response of a fluorescent sample to a frequency modulated illuminating light. The photodegradation rate is obtained by interpreting the frequency domain measurements in terms of traditional impedance concepts. In the measurements described in this paper, a focused laser beam is used to illuminate a sample of slowly flowing fluorescent solution. The laser beam is assumed to have a Gaussian power distribution hence illumination is spatially non-uniform in the region of interest. The photochemical reaction rates depend on power, so they will also vary with the position in the beam. However in the case of photodegradation of fluorophores, the measurement of the resulting decrease in fluorescence is given in terms of the radiation emitted from the entire illuminated region. In this work we present a mathematical description of the time evolution of the fluorescence response integrated over a non-uniformly illuminated domain. As a result of our analysis, an experimentally accessible and tractable mathematical model Eq. (19) and Eq. (30) is obtained from a more fundamental description given by Eq. (4) and Eq. (5). The model is used to create a functional form for fitting experimental measurements from a lock-in amplifier.

## 1. Introduction

**Fig. 1:**
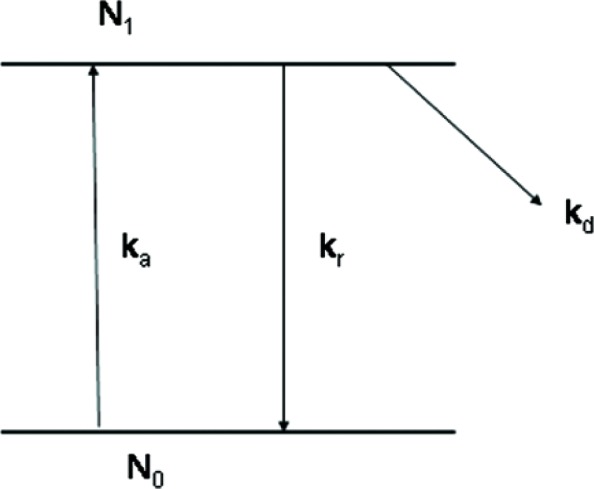
A graphical representation of the components included in the kinetic model discussed in the text. The rate constant *k_d_*, represents photodegradation that leads to non-fluorescent species. The rate constants *k_a_* and *k_r_* represent absorption and relaxation to the ground state respectively. *N*_0_ and *N*_1_ represent the populations of the ground and excited states.

The photodegradation of fluorophores is an important process that determines the accuracy of many biological assays [1], the efficacy of photodynamic therapy [2], and the phototoxicity of drugs. The measurement of the apparent rate of photodegradation was traditionally performed by measuring the decrease in fluorescence emission under constant illumination [3]. Recently illumination consisting of short pulses has been used to examine the decay of photochemical reaction products [4]. The time resolved measurements are difficult to interpret since the photo-degradation process usually involves multiple time scales. The frequency domain measurement technique looks at the response for each frequency of modulation of the illuminating light. Harmonic equations are simpler to solve and, thus, interpretation of frequency domain measurements can be performed in terms of traditional impedance concepts [5]. In the measurements described below, a focused laser beam is used to illuminate the sample. The power distribution of a Gaussian laser beam is strongly dependent on spatial location; therefore, the rates of photochemical reactions, which depend on power, will also depend on location in the beam. Since absorption of radiation leads to local heating and concentration gradients, convective and diffusive mass transport will be present in the vicinity of the focused beam. A slow flow is imposed on the fluorophore solution in order to dominate and thus minimize the effect due to uncontrollable convective and diffusive mass transport. In the case of photodegradation of fluorophores, measurement of the resulting decrease in fluorescence usually measures the radiation emitted from the entire illuminated region. Therefore an analysis method has to be developed which describes the time evolution of the integrated fluorescence response from a nonuniformly illuminated region. In previous work [6] a frequency domain measurement technique was interpreted with a model which assumed a uniform beam profile. This work extends the model to a laser beam with a Gaussian profile. In it we derive an experimentally accessible mathematical model Eq. (19) and Eq. (30) from first principles Eqs. (4) and (5). A dramatic simplification of these equations is a consequence of the strongly disparate time scales in the kinetics imposed by the slow rate of degradation relative to the relaxation and absorption rates The first section contains a motivation and presentation of the equations that are taken as a fundamental description of the experimental setting (also see Fig. 1). The equations are based on a two-state model with excitation and relaxation. In Sec. 2, a perturbation scheme employing the disparate time scales is used to reduce the system of two partial differential equations, describing the kinetic process to a single partial differential equation Eq. (14) for the total fluorophore population (to leading order in the perturbation parameter). The biggest reduction in mathematical complexity comes from writing the total measured fluorescence in terms of the total fluorophore population averaged over the Gaussian light beam distribution. This formula is introduced in Sec. 3, Eq. (9) and there follows the derivation of a single ordinary equation for the time evolution of the spatial average Eq. (30) that completes the model. The mathematical arguments that justify the manipulations performed in the main body of the paper can be found in the appendices. Section 4 discusses the time independent case and a functional form for fitting experimental measurements from a lock-in amplifier. The form, obtained from fitting values predicted from the model, shows good agreement with actual data. In a forthcoming paper, a harmonic approximation of the solution of the model equations will be developed. It will be possible then to express the fit parameters in terms of the physical parameters in the experiment.

## 2. Kinetic Equations

As a starting point, consider a model for reactions at a fixed point in space. This simplified model will be used to motivate the details of the analysis. Assuming no mass transfer from surrounding fluid, Fig. 1 shows a two state model where absorption occurs from the ground state with rate *k_a_*, and the excited state population of size *N*_1_, relaxes back to the ground state with a rate *k_r_*. Here *N*_0_ is the population of the ground state molecules. Intact fluorophores change into non-fluorescent species with a rate *k_d_*. The kinetics are summarized by a set of equations

$$\begin{array}{l} \frac{dN_{0}}{dt}=-k_{a}N_{0}+k_{r}N_{1}\\ \frac{dN_{1}}{dt}=k_{a}N_{0}-k_{r}N_{1}-k_{d}N_{1}. \end{array}$$ (1)

To interpret *k_d_*, Eq. (1) has to be supplemented by a model of the photochemical processes that are responsible for the conversion of the fluorophores into non-fluorescent species [6]. In the present manuscript, there will be no attempt to interpret the rate constant *k_d_*. The discussion below will focus on two problems inherent in using Eq. (1) to describe real experimental conditions. The first problem is that the laser beam has a spatial intensity distribution leading to spatial variation of the absorption rate. The second problem is that convection and diffusion currents will change the local concentration of molecules in an uncontrolled manner. The second problem will be reduced significantly by introducing a flow that will change the concentration of fluorophores in a predictable way.

The absorption rate depends on the power density of the incident light. The absorption rate will be written as *k_a_* = *σ_a_I* [7], where *σ_a_*(cm^2^) is the molecular absorption cross section, and *I* is the incident photon flux (1/s cm^2^). During the experiment, the laser irradiance *P*(W/cm^2^) is measured. The irradiance *P* can be converted into a photon flux, *I*, by dividing *P* by the energy per photon. Explicitly, *I* = *P_c_P* where the conversion factor is given by Pc=λnhc. Here *λ* is the wavelength, *n* is the index of refraction, *h* is Planck’s constant, and *c* is the speed of light in vacuum. This yields the result *k_a_* = *σ_a_P_c_P* = *k_c_P*. The constant *k_c_* relates the laser irradiance to the molecular absorption rate.

**Fig. 2:**
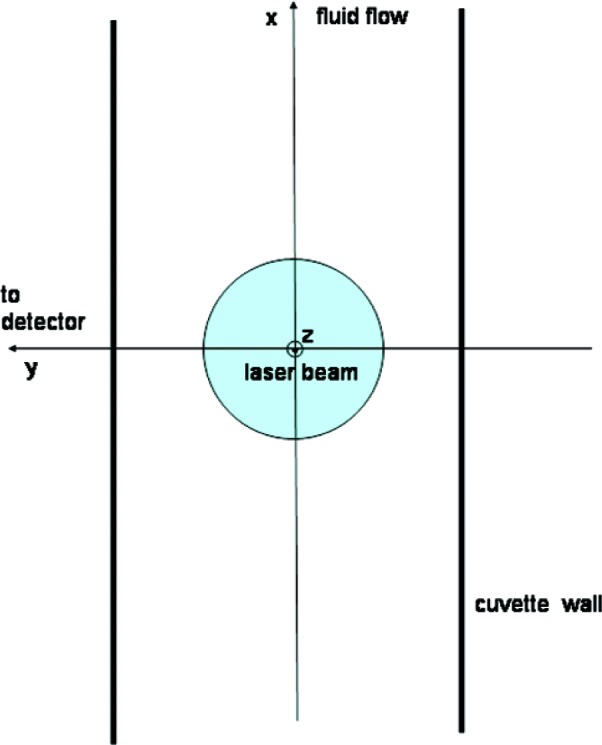
The geometric arrangement of cuvette walls (thick lines), laser beam propagating in the *z* direction (out of the plane of the paper), the fluid velocity in the *x* direction, and the detector located on the *y* axis. The shaded circle represents the Gaussian profile of the laser beam.

Figure 2 shows the geometric relation of the laser beam, the cuvette holding the sample, the flow, and the detector. The laser beam is incident along the z axis which points out of the plane of the paper. The irradiance of the modulated beam that illuminates the sample will be written as:

$$P(x,y,t)=P_{0}(x,y)+\Delta P(x,y)\cos(\omega t).$$ (2)

*P*_0_(*x, y*) is the time independent component, and Δ*P*(*x, y*) is the amplitude of the modulated component, *ω* is the radial modulation frequency, $\frac{\omega }{2\pi }$ is the frequency in Hz, and *t* is time. The beam will be assumed to have a Gaussian profile [8].

$$\begin{array}{l} f(x,y)=\frac{1}{\pi w^{2}}\exp\left(-\frac{x^{2}+y^{2}}{w^{2}}\right)\\ P_{0}(x,y)=P_{0}f(x,y)\\ \Delta P(x,y,t)=P_{1}f(x,y)\cos(\omega t) \end{array}$$ (3)

where *P*_0_ is the total power (watts) of the laser beam and *w* is the width of the beam. The function *f* (*x, y*) is defined such that its integral over all the *x, y* plane is normalized to 1. The time independent component and the modulation amplitude have the same spatial dependence given by *f* (*x, y*). Clearly in this situation, the absorption rate will have a strong spatial dependence. Consequently reaction products may have concentration gradients and uneven heating may cause thermal mass transfer. The kinetic model of photodegradation, given by Eq. (1), will be extended to include a spatially dependent absorption rate and a constant flow of the solution containing the reaction product. It will be assumed that the flow dominates all mass transport (diffusive and convective) into and out of the illuminating region and provides a well defined initial concentration of fluorophore in the illuminated region.

To account for the spatial variation of absorption and the flow of fluid carrying fluorophores past the laser light, Eq. (1) is modified to,

$$\begin{array}{l} \frac{\partial N_{0}(x,y,t)}{\partial t}+v\frac{\partial N_{0}(x,y,t)}{\partial x}=-k_{a}(x,y,t)N_{0}(x,y,t)+k_{r}N_{1}(x,y,t)\\ \frac{\partial N_{1}(x,y,t)}{\partial t}+v\frac{\partial N_{1}(x,y,t)}{\partial x}=k_{a}(x,y,t)N_{0}(x,y,t)-k_{r}N_{1}(x,y,t)-k_{d}N_{1}(x,y,t) \end{array}$$ (4)

Here *k_a_* (*x, y, t*) = *k_c_P*(*x, y, t*). The velocity of the fluid, denoted by v is assumed to be a constant and in the direction of the *x* axis in Fig 2. Note in contrast to Eq. (1) the absorption rate is now a function of space and time. To completely specify the solution of these equations, the number of particles flowing into the entrance of the apparatus must be specified and the number of particles at the beginning of the observation period (*t* = 0) must also be given.

$$\begin{array}{l} N_{0}(-L,y,z,t)=N^{0},N_{1}(-L,y,z,t)=0\;\text{for}\;t\geq 0\\ N_{0}(x,y,z,0)=N^{0},N_{1}(x,y,z,0)=0\;\text{for}\;x\geq -L \end{array}$$ (5)

Equation (5) states that prior to entering the laser beam there is no change in the populations of the fluorophore states. The distance L is some length along the *x* axis where the laser beam intensity is effectively zero. Eq. (5) also gives the initial conditions at *t* = 0 when the laser beam is turned on.

## 3. Perturbation Analysis of Kinetic Equations

The faster processes, absorption and relaxation dominate the time variation at least initially. Returning to the model for the fixed position in Eq. (1), this suggests that after an initial transient the quasi-equilibrium condition holds,

$$N_{1}\approx \frac{k_{a}}{k_{r}}N_{0}.$$

Substitution of this condition into Eq. (1) results in a single equation for the total fluorophore population *N* = *N*_0_ + *N*_1_.

$$\frac{dN}{dt}=\frac{-k_{d}k_{a}}{k_{r}+k_{d}}N.$$

In this section this reduction is extended to the case of non-uniform illumination and is made rigorous. Here the coupled pair of partial differential equations, Eq. (4), will reduce to a single partial differential equation Eq. (14), accurate to first order in a perturbation expansion. Justification of the operations leading to these simplifications can be found in Appendix A.

The absorption and the excited state relaxation rates are usually of the order 10^8^ s^−1^, while the value of *k_d_* is less than 10^3^ s^−1^. The constants in Eq. (4) can therefore be re-scaled as $k_{a}={\bar{k}}_{a}/\varepsilon$, $k_{r}={\bar{k}}_{r}/\varepsilon$ where *ε* = 10^−8^ and ${\bar{k}}_{a}$, ${\bar{k}}_{r}$ are of the order of 1. Equation (4) can then be rewritten as,

$$\begin{array}{l} \varepsilon \left(\frac{\partial N_{0}(x,y,t)}{\partial t}+v\frac{\partial N_{0}(x,y,t)}{\partial x}\right)=-{\bar{k}}_{a}(x,y,t)N_{0}(x,y,t)+{\bar{k}}_{r}N_{1}(x,y,t)\\ \varepsilon \left(\frac{\partial N_{1}(x,y,t)}{\partial t}+v\frac{\partial N_{1}(x,y,t)}{\partial x}\right)=-{\bar{k}}_{a}(x,y,t)N_{0}(x,y,t)-{\bar{k}}_{r}N_{1}(x,y,t)-\varepsilon k_{d}N_{1}(x,y,t) \end{array}$$ (6)

We will treat *N*_0_ (*x, y, t*,) and *N*_1_ (*x, y, t*) as functions of *ε*. Let ${\hat{N}}_{0}(x,y,t)={N_{0}(x,y,t,\varepsilon )|}_{\varepsilon =0}$ and ${\hat{N}}_{1}(x,y,t)={N_{1}(x,y,t,\varepsilon )|}_{\varepsilon =0}$ be the leading terms for *N*_0_ (*x, y, t, ε*) and *N*_1_ (*x, y, t, ε*) respectively in a perturbation expansion for *ε* > 0 and large time *t*.

$$\begin{array}{l} N_{0}(x,y,t,\varepsilon )={\hat{N}}_{0}(x,y,t)+\varepsilon {\hat{N}}_{01}(x,y,t)+O(\varepsilon^{2})\\ N_{1}(x,y,t,\varepsilon )={\hat{N}}_{1}(x,y,t)+\varepsilon {\hat{N}}_{11}(x,y,t)+O(\varepsilon^{2}). \end{array}$$ (7)

The validity of the expansion in *ε* is discussed further in Appendix A. Substituting Eq. (7) into Eq. (6) and equating coefficients of equal powers of *ε* on both sides of the equation gives for *ε* = 0

$$\begin{array}{l} 0=-{\bar{k}}_{a}(x,y,t){\hat{N}}_{0}(x,y,t)+{\bar{k}}_{r}{\hat{N}}_{1}(x,y,t)\\ 0={\bar{k}}_{a}(x,y,t){\hat{N}}_{0}(x,y,t)-{\bar{k}}_{r}{\hat{N}}_{1}(x,y,t). \end{array}$$ (8)

This implies that as *ε* → 0

N^1(x,y,t)=(ka(x,y,t)kr)N^0(x,y,t). (9)

Thus the relation between the populations of ground and excited states holds in this approximate sense for all larger times. (This is not valid for *t* ≈ 0. When the illumination is turned on, there is an initial transient period where the populations are changing rapidly and therefore where the left hand side of Eq. (6) is not proportionate to *ε*.) Equating the coefficients of the first power of *ε* gives:

$$\begin{array}{l} \frac{\partial {\hat{N}}_{0}(x,y,t)}{\partial t}+v\frac{\partial {\hat{N}}_{0}(x,y,t)}{\partial x}={\bar{k}}_{r}{\hat{N}}_{11}(x,y,t)-{\bar{k}}_{a}(x,y,t){\hat{N}}_{01}(x,y,t)\\ \frac{\partial {\hat{N}}_{1}(x,y,t)}{\partial t}+v\frac{\partial {\hat{N}}_{1}(x,y,t)}{\partial x}={\bar{k}}_{a}(x,y,t){\hat{N}}_{01}(x,y,t)-{\bar{k}}_{r}{\hat{N}}_{11}(x,y,t)-{\bar{k}}_{a}{\hat{N}}_{1}(x,y,t). \end{array}$$ (10)

Letting $N(x,y,t)={\hat{N}}_{0}(x,y,t)+{\hat{N}}_{1}(x,y,t)$, be the total fluorophore population in the zero order approximation, we can add the two parts of Eq. (10) and obtain

$$\frac{\partial N(x,y,t)}{\partial t}+v\frac{\partial N(x,y,t)}{\partial x}=-k_{d}{\hat{N}}_{1}(x,y,t).$$ (11)

The right side of Eq. (11) depends on the zeroth order approximation to the population of excited state. Using Eq. (9) and the definition of the total fluorophore population it is possible to obtain the relation

$${\hat{N}}_{1}(x,y,t)=\frac{k_{a}(x,y,t)}{k_{r}+k_{a}(x,y,t)}N(x,y,t)$$ (12)

which is correct to zero order in *ε*. Substituting Eq. (12) into the right hand side of Eq. (11) gives the following equation for *N* (*x, y, t*) valid up to first order in *ε*

$$\frac{\partial N(x,y,t)}{\partial t}+v\frac{\partial N(x,y,t)}{\partial x}=\frac{-k_{d}k_{a}(x,y,t)}{k_{r}+k_{a}(x,y,t)}N(x,y,t).$$ (13)

Equation (10) has been rewritten as a kinetic equation for the total fluorophore number valid to the first order in *ε*. Equation (13) can be rewritten further by setting *b* = *k_c_*/*k_r_*, *η* = *k_d_b*. Eq. (13) becomes

$$\frac{\partial N(x,y,t)}{\partial t}=\frac{-\eta P(x,y,t)}{1+bP(x,y,t)}N(x,y,t)-v\frac{\partial N(x,y,t)}{\partial x}$$ (14)

The boundary condition for *N*(*x, y, t*) at *x* = − *L* is inherited from those imposed on *N*_0_ and *N*_1_ at *x* = − *L* in Eq. (5)

$$N(-L,y,t)=N^{0}.$$ (15)

The initial condition at *t* = 0 is *N*(*x, y*, 0) = *N*^0^ since no reaction takes place prior to turning on the illumination.

The last term in Eq. (14) gives the flow related flux of fluorophores into the illuminating region as a function of position. The flow of fluorophores into the volume illuminated by the laser beam is assumed to be uniform over the entire volume. The photodegradation depends on the

## 4. Spatial Averaging and the Fluorescent Signal

The measured fluorescence signal per unit distance in the *z* direction is given by

$$F(t)=Ak_{rad}\iint_{R}N_{1}(x,y,t)dxdy$$ (16)

where *A* represents the characteristics of the optical measurement instrument, *k*_rad_ is the rate of radiative decay of the excited state population, and *N*_1_(*x, y, t*) is the population of the excited state. (The radiative decay contributes to the overall relaxation rate given by *k_r_*). The population of the excited state can be approximated by Eq. (12) leading to

$$F(t)=Ak_{rad}\iint_{R}\frac{bP(x,y,t)}{1+bP(x,y,t}N(x,y,t)dxdy.$$ (17)

The symbol *b* was defined previously. Equation (17) together with Eq. (14) constitute a description of the measurement valid to first order in *ε*. Equation (17) suggests that instead of finding the solution *N*(*x, y, t*) at every spatial point it may be better to find the variation of the averaged concentration defined by Eq. (18)

$$\langle N(t)\rangle \equiv \iint_{R}f(x,y)N(x,y,t)dxdy.$$ (18)

The average is performed with a weighting factor equal to the spatial distribution of the laser irradiance. The weighting factor provides a measure of the contribution of fluorophores at each point in space to the total fluorescence signal. It will be shown below that Eq. (14) can be converted into an equation for 〈*N*(*t*)〉 as defined by Eq. (18), and that the fluorescence signal can be written as

$$F(t)=Ak_{rad}b\left[P(t)\langle N(t)\rangle -b\alpha {\left(P\left(t\right)\right)}^{2}\langle N(t)\rangle \right]$$ (19)

where to a very good approximation *α* is a constant and *P*(*t*) = *P*_0_ + *P*_1_cos(*ω t*) ≡ *P*_0_ + Δ(*t*). For actual computations, the constant *α* is evaluated in terms of the time independent solution of Eq. 14. In this section, an ordinary differential equation for the time evolution of 〈*N*(*t*)〉 will be derived (Eq. (30)). The fluorescent signal can therefore be obtained (with small error) by solving Eq. (30) and then using Eq. (19). Together these equations represent an experimentally accessible mathematical model of the fluorescent signal under excitation by a focused laser beam.

To find an equation for 〈*N*(*t*)〉, first multiply Eq. (14) by 1 + *bP*(*x, y, t*) and obtain (below *r* represents the coordinates *x,y*).

$$\begin{array}{l} \left(1+bP(r,t))\frac{\partial N(r,t)}{\partial t}=-\eta P(r,t)N(r,t\right)\\ -v(1+bP(r,t))\frac{\partial N(r,t)}{\partial x}. \end{array}$$ (20)

Multiply Eq. (20) by *f* (*x, y*) and integrate over the beam cross section region, R. Each term will be treated in detail. To simplify the notation integrals in the *x–y* plane will be represented by a single integral in *r* = (*x, y*) with *dr* = *dxdy*. The left side of Eq. (20) becomes

$$\int_{R}f(r)(1+bP(r,t))\frac{\partial N(r,t)}{\partial t}dr$$

which can be expanded to

$$\int_{R}\frac{\partial N(r,t)}{\partial t}f(r)dr+b\int_{R}P(r,t))\frac{\partial N(r,t)}{\partial t}f(r)dr.$$

Inserting the explicit form of the power function we get

$$\begin{array}{l} \frac{\partial \langle N(t)\rangle }{\partial t}+b\int_{R}(P_{0}+\Delta (t))f(r)\frac{\partial N(r,t)}{\partial t}f(r)dr\\ =\frac{\partial \langle N(t)\rangle }{\partial t}+b(P_{0}+\Delta (t))\int_{R}f(r)\frac{\partial N(r,t)}{\partial t}f(r)dr\\ =\frac{\partial \langle N(t)\rangle }{\partial t}+b(P_{0}+\Delta (t))\frac{\partial }{\partial t}\int_{R}f(r)N(r,t)f(r)dr. \end{array}$$

The second part of the above equation will be rewritten by introducing the quantity,

$$\bar{\alpha }(t)\equiv \frac{\int_{R}f(r)f(r)N(r,t)dr}{\int_{R}f(r)N(r,t)dr}.$$ (21)

The final result for the left side of Eq. (20) after multiplication and integration is therefore

$$\frac{\partial \langle N(t)\rangle }{\partial t}+b(P_{0}+\Delta (t))\frac{\partial }{\partial t}\left\{\bar{\alpha }(t)\int_{R}f(r)N(r,t)dr\right\}$$ (22)

(Note that the definition given in Eq. (21) will be used in deriving Eq. (19)).

The first term on the right side of Eq. (20) after the operations becomes

$$\begin{array}{l} -\eta \int_{R}P(r,t)N(r,t)f(r)dr\\ =-\eta (P_{0}+\Delta (t))\int_{R}f(r)N(r,t)f(r)dr\\ =-\eta \bar{\alpha }(t)(P_{0}+\Delta (t))\langle N(t)\rangle \end{array}$$ (23)

where $\bar{a}$ is defined in Eq. (21). The second term on the right side of Eq. (20) becomes, after multiplication and integration,

$$\begin{array}{l} -v\int_{R}(1+bP(r,t))\frac{\partial N(r,t)}{\partial x}f(r)dr\\ =-v\int_{R}\frac{\partial N(r,t)}{\partial x}f(r,t)dr\\ -vb(P_{0}+\Delta (t))\int_{R}f(r)\frac{\partial N(r,t)}{\partial x}f(r)dr. \end{array}$$

To treat the second term of the above equation, we introduce a second function defined by

$${\bar{\alpha }}^{\prime }(t)\equiv \frac{\int_{R}f(r)f(r)\frac{\partial N(r,t)}{\partial x}dr}{\int_{R}f(r)\frac{\partial N(r,t)}{\partial x}dr}.$$ (24)

The second term of the right hand side of the transformed Eq. (20) now reads

$$-v(1+b(P_{0}+\Delta (t)){\bar{\alpha }}^{\prime }(t))\int_{R}f(r)\frac{\partial N(r,t)}{\partial x}dr.$$ (25)

In Appendix B we will show that the functions $\bar{\alpha }$ and ${\bar{\alpha }}^{\prime }(t)$ are, up to a small error, independent of *t* and can be replaced by constants *α* and *α*′ respectively. The error introduced into equations Eq. (21), Eq. (23), and Eq. (25) by this approximation is discussed in Appendix C.

Collecting terms, Eq. (20) can be written after multiplication by *f* (*x, y*) and integration, up to small varying functions of *t*, as

$$\begin{array}{l} (1+b\alpha (P_{0}+\Delta (t)))\frac{\partial \langle N(t)\rangle }{\partial t}\\ =-\eta \alpha (P_{0}+\Delta (t))\langle N(t)\rangle \\ -v(1+b\alpha^{\prime }(P_{0}+\Delta (t)))\langle \frac{\partial N}{\partial x}\rangle (t) \end{array}$$ (26)

where

$$\langle \frac{\partial N}{\partial x}\rangle (t)=\int_{R}f(r)\frac{\partial N(r,t)}{\partial x}dr.$$ (27)

The final step in the derivation of an equation for 〈*N*(*t*)〉 consists of finding an approximation to the weighted average spatial derivative in Eq. (27). For small widths w the major contribution to the integral comes from a region in the vicinity of the beam which we define by 0 ≤|*x*|,|*y*|≤ *κ w* where we may choose the constant *κ*, 0 ≤ *κ* ≤ 3. If we replace the partial derivative in the integrand by a backwards difference quotient with step *h* = − *κ w* the value of *N* at the left endpoint of the interval is *N*^0^ (to a good approximation). Thus for *w* small enough we have,

$$\langle \frac{\partial N}{\partial x}\rangle (t)=\frac{\langle N(t)\rangle -N^{0}}{\kappa w}+o(1)$$

where *o*(1)is a term that tends to zero as *w* → 0. To guarantee an error that is smaller than the dominant terms of Eq. (26) we have to require that *w* is sufficiently small and *κ* is large. In fact experimental conditions govern the actual choice of *w*. The following heuristic argument governed the choice of *κ*.

We observed that in the vicinity of the origin (*x* = 0, *y* = 0) the spatial derivative of the time independent solution, *N*(*x, y*) discussed below, has a functional form which is almost identical to the beam distribution function *f* (*x, y*) as shown by the two overlapping curves in Fig. 4. The difference between − *f* (*x, y*) and ∂*N*(*x, y*)/∂*x* (both normalized by dividing by the maximum of their respective absolute values) is magnified 50 times and shown in Fig. 4 by the upper curve. The weighted average of the un-normalized beam distribution function *π* · *width*^2^ *f* (*x, y*) is equal to 0.5. Therefore the weighted average of the spatial derivative will be approximately equal to 0.5 times the value at the origin

$$\langle \frac{\partial N}{\partial x}\rangle (t)=\int_{R}f(x,y)\frac{\partial N}{\partial x}(x,y,t)dxdy\approx \frac{1}{2}\frac{\partial N}{\partial x}(0,0,t).$$ (28)

The derivative at the origin can be approximated in the usual way by the difference of the function *N*(*x, y, t*) evaluated at two points spanning the origin and divided by the distance between the two points. However for the purpose of this analysis a more useful approximation uses the difference 〈*N* (*t*)〉 − *N*^0^ divided by an effective distance which reproduces the value of the derivative. For the case of the Gaussian beam, the best estimate of the derivative at the origin was found (using the time independent solution) to be (〈*N* (*t*)〉 − *N* ^0^)/w*0.637. This yields the following approximate expression for the average spatial derivative given in Eq. (28).

$$\langle \frac{\partial N}{\partial x}\rangle (t)=\frac{\langle N(t)\rangle -N^{0}}{1.274\cdot w}$$ (29)

Using Eq. (29), the approximate kinetic equation for 〈*N* (*t*)〉 becomes

$$\begin{array}{l} \frac{d}{dt}\langle N(t)\rangle =\frac{-\eta \alpha (P_{0}+\Delta (t))}{1+b\alpha (P_{0}+\Delta (t))}\langle N(t)\rangle \\ \;-\frac{v}{1.274\cdot w}\cdot \frac{\left(1+b\alpha^{\prime }(P_{0}+\Delta (t))\right)}{\left(1+b\alpha (P_{0}+\Delta (t))\right)}(\langle N(t)\rangle -N^{0}) \end{array}$$ (30)

where Δ(*t*) = *P*_1_ cos(*ωt*).

## 5. Validation of the Model Equations and Their Derivation

This section discusses the time independent form of Eq. (30) (see Eq. (31). Since an analytical form of the solution exists for the time independent case of Eq. (14), direct comparisons of the fluorescent signal obtained from the time independent version of Eq. (30) are possible. In both cases the fluorescence signal is obtained from Eq. (19) (using the steady state solutions of Eq. (14) and Eq. (30). A second important reason for considering the time independent solution is that the spatial average in Eq. (18) is dominated by the spatial average of the time independent solution itself. As a consequence, the time varying functions *α* (*t*) and *α*′ (*t*) can be replaced by constants calculated from the time independent solution (see B 9 in Appendix B).

The time independent form of Eq. (14) corresponds to a condition of constant illumination, constant flow, and constant initial fluorophore concentration.

$$\frac{\eta P_{0}f(x,y)}{1+bP_{0}f(x,y)}N(x,y)=-v\frac{\partial N(x,y)}{\partial x}$$ (31)

Figure 2 shows the geometrical arrangement of the flow channel, the laser beam, and the detector. The laser beam is along the *z* direction, the detector along the *y* axis, and the flow takes place along the *x* axis. The channel dimensions are d *y* = 0.4 cm along the *y* axis, d *z* = 1 cm along the *z* axis, and 5 cm along the *x* axis. The velocity of the fluid in the center of the channel is set to 0.025 cm/s. No uncertainties are given since these values will be used to model the response of the system. The width of the Gaussian laser beam is set to *w* = 50 · 10^−4^ cm. The laser power is *P*_0_ = 0.03 W, the vacuum wavelength is *λ* = 488 · 10^−7^ cm. The number of photons per Joule, *P*_c_, is calculated by *P*_c_ = *λ n*/*hc* = 3.314 · 10^21^ J^−1^, where *n* = 1.35 is the index of refraction of water and *hc* is the product of Planck constant and the speed of light. The extinction coefficient is set to *ε* = 82000 L/cm mol, yielding a cross section *σ* = 1000*ε*/0.4343 *N_A_* = 3.135 · 10^−16^ cm^2^ where *N_A_* is the Avogadro’s number. The average absorption rate is given by $k_{a}=\sigma P_{c}P_{0}/\surd \bar{\pi }width=3.97\cdot 10^{5}s^{-1}$, and the relaxation rate is given by the inverse of the measured lifetime of the excited state, *k_r_* = 1/*τ* = 3.33 · 10^8^ s^−1^. The photodegradation rate *k_d_* is set to 170 s^−1^. Using these values it is possible to calculate the parameters that enter into Eq. 31,*b* = *σ P_c_*/*k_r x_* = 3.117 · 10^−6^ cm^2^ s/J and *η* = *k_d_b* = 5.3 · 10^−4^ cm^2^/J.

**Fig. 3:**
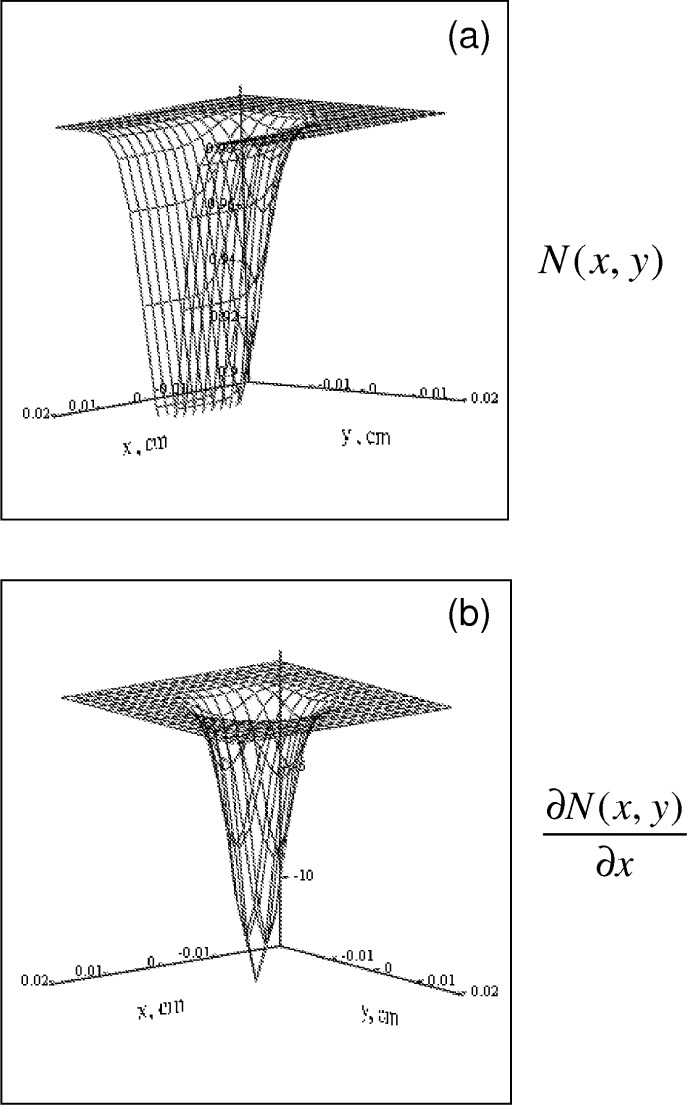
(a) The steady state solution of Eq. (31) given by Eq. (32). The physical parameters that enter into the equation are discussed in the text. The function *N*(*x, y*) is normalized to 1. The laser beam is incident along the normal to the *x-y* plane. As expected, the number of intact fluorophores decreases with increasing laser beam intensity. The decreased concentration of intact fluorophores persists along the *x* axis due to the imposed flow. (b) The steady state *x*-derivative of the function *N*(*x, y*). The derivative is finite along the profile of the laser beam.

**Fig. 4:**
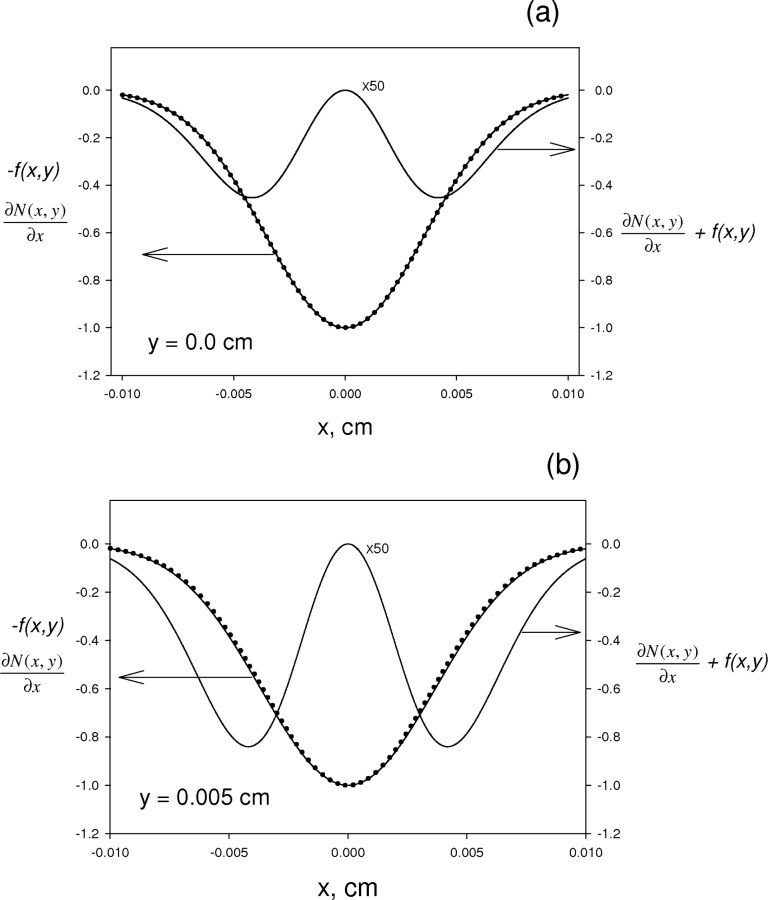
The correspondence between the Gaussian beam profile function − *f* (*x, y*), shown by the solid line, and the steady state function $\frac{\partial N(x,y)}{\partial x}$, shown by the dotted line. Both functions are normalized by their maximum values in order to emphasize functional similarities. The difference between the two functions, magnified 50 times, is shown by the curve with two valleys. The graphs in Fig. 4a are for *y* value of 0 cm, and the graphs in Fig. 4b are for *y* value of 0.005 cm.

The solution of Eq. 31 is written as

$$\begin{array}{l} N(x,y)=Ae^{c(x,y)}\\ c(x,y)=\int_{-L}^{x}\frac{-1}{v}\frac{\eta P_{0}f(x^{\prime },y)}{1+bP_{0}f(x^{\prime },y)}{dx}^{\prime } \end{array}$$ (32)

where *L* = 0.02. Equations (21) and (24) can be used to calculate *α* = 6324 and *α′* = 8462, respectively, using Eq. (32). Note that *A* is a constant which is set to 1. Figure 3 shows the resulting *N*(*x, y*) for the parameters given above. The solution given by Eq. (32) can be used to predict the time independent fluorescence signal by substituting it into Eq. (19). The result can be compared to fluorescence calculated from the steady state of the approximate kinetic Eq. (30), i.e.,

〈N〉steady state=11+ηαP0v1.26w(1+bα′P0)N0. (33)

The two values of the fluorescent signal (FS) agree to within 1 % up to 0.1 W incident power. The approximate fluorescence signal given by Eq. (30) and Eq. (33) is almost indistinguishable from the result given by Eq. (32). It is expected that the time dependent solutions will also be described correctly by the approximate Eq. (30) which together with Eq. (19) will be taken as the fundamental description of the photodegradation process occurring in the focused laser beam. Finally the steady state solution was used to find the effective distance to use in the estimate of the average spatial derivative given by Eq. (29). The exact value of the average spatial derivative and the estimated value using Eq. (29) are − 3.932 cm^− 4^ and − 3.906 cm^−4^ respectively. Thus Eq. (29) should provide a reasonable estimate of the average spatial derivative even in the time dependent case.

The formal time dependent solution of Eq. (30) can be written as (see for example http://tutorial.math.lamar.edu/terms.aspx)

$$\langle N(t)\rangle =N^{0}e^{-\int_{0}^{t}p(t^{\prime })dt^{\prime }}(1+\int_{0}^{t}e^{\int_{0}^{t}p(t^{\prime \prime })dt^{\prime \prime }}G(t^{\prime })dt^{\prime })$$ (34)

where

$$\begin{array}{l} p(t)=\frac{\eta \alpha P(t)}{1+b\alpha P(t)}+\frac{v}{1.274w}\frac{1+b\alpha^{\prime }P(t)}{1+b\alpha P(t)}\\ G(t)=\frac{v}{1.274w}\frac{1+b\alpha^{\prime }P(t)}{1+b\alpha P(t)}. \end{array}$$ (35)

For the case of low power, the approximations *bα P*(*t*) « 1, *bα′ P*(*t*) « 1, and a laser beam power given by *P*(*t*) = *P*_0_ + *P*_1_ cos (*ω t*) yield an approximate solution given by Eq. (36).

$$\begin{array}{l} \langle N(t)\rangle =N^{0}e^{-(\eta \alpha P_{0}+v/1.274w)t}e^{-\frac{\eta \alpha P_{1}}{\omega }\sin(\omega t)}•\\ \left(1+\frac{v}{1.274w}\int_{0}^{t}e^{(\eta \alpha P_{0}+v/1.274w)t^{\prime }}e^{\frac{\eta \alpha P_{1}}{\omega }\sin(\omega t^{\prime })}dt^{\prime }\right). \end{array}$$ (36)

**Fig. 5:**
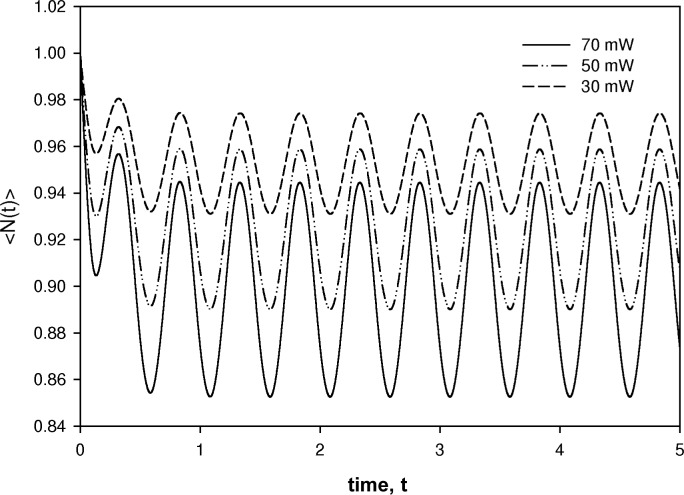
The time dependent numerical solutions of Eq. (30). Each curve corresponds to a different power of the laser beam. The initial value is set to 1 in all cases. After an initial transient, the average concentration of intact fluorophores oscillates with the modulation frequency. The amplitude of the oscillation increases with increasing laser power.

**Fig. 6:**
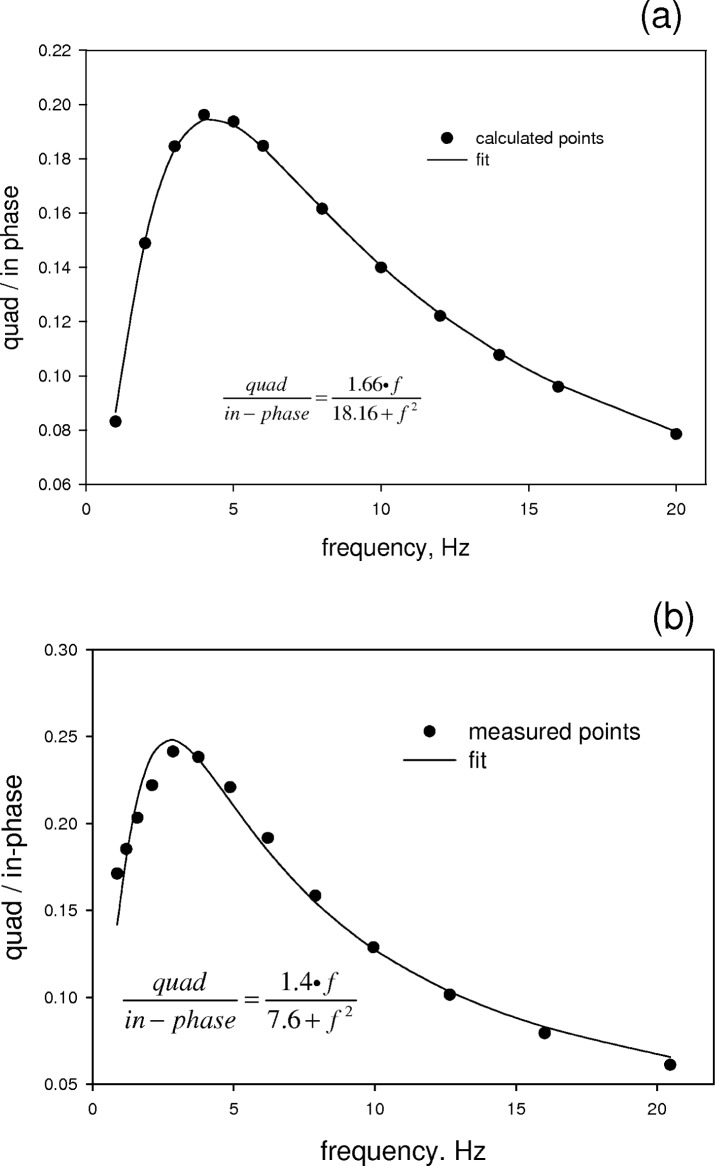
(a) The time dependent solutions, such as shown in Fig 5, were inserted into Eq. (19) to model the detected fluorescence signal. The fluorescence signal was inserted into a digital model of a two phase lock-in amplifier (discussed in the text). The ratio of the outputs of the quadrature and in-phase lock-in amplifiers is shown by the solid circles. The solid line is a best fit to a function *R*(*f*) = (a* *f*)/(*b* + *f*^2^), where *f* is the modulation frequency and *a* and *b* are parameters. (b) The solid circles show the measured response of a flowing fluorescein solution in a focused laser beam with a wavelength of 488 nm. The solid line gives the best fit to the function *R*(*f*) given in (a).

Figure 5 shows the time dependent solution at three values of the laser power for a modulation frequency of Hz. The other parameters were taken to be the same as in the case of the steady state solution. The salient features of the time dependent solution are a transient at *t* = 0 and a modulation of the intact fluorophore concentration at the driving frequency of the laser. As expected the average level of intact fluorophores decreases with increasing average power, and the amplitude of the modulation increases with increasing modulation amplitude. The approximate time dependent solution given by Eq. (36) was used to obtain the fluorescence signal given by Eq. (19). The fluorescence signal was detected by a computer model of a lock-in amplifier consisting of an in-phase and quadrature multipliers followed by low pass filters. The low pass filters were implemented by Fourier analyzing the signal, setting to zero all coefficients corresponding to non zero frequencies, and then performing an inverse Fourier transform. The solid circles in Fig. 6a show the ratio of the detected quadrature and in-phase components at different modulation frequencies. Most of the parameters used in Eq. (36) were the same as in the steady state calculation except for *width* = 10 µm, *P*_0_ = 50 mW, and *k_D_* = 600 s^−1^. The solid line in Fig. 6a shows a fit to the calculated points of a function given by *R*(*f*) = (a* *f*)/(*b* + *f*^2^), where *f* is the modulation frequency and *a* and *b* are fit parameters. Clearly the calculated response and *R*(*f*) form an excellent match. The solid points in Fig. 6b show experimentally measured values of the ratio of quadrature and in-phase response. The solid line in Fig 6b is a fit to the function *R*(*f*). The match is reasonable. Thus there is a strong indication that the measured response can be represented by the function *R*(*f*), and that the fit parameters provide information about the various physical constants of the experiment. To elucidate the dependence of the fit parameters on the physical constants, it is best to use analytical solutions to Eq. (30) and obtain the analytical form of the response function *R*(*f*). This task is carried out in a forthcoming paper.

We close this section with a brief description of the mathematical justification for the time dependent case. The reduction of the original system Eq. (4) and Eq. (5) to a single partial differential equation depended on the use of a multiple time scale perturbation expansion. The validity of the technique used for this problem (matched asymptotic expansions) is discussed in Appendix A. By spatially averaging the partial differential equation we derive an ordinary differential equation for the averaged reactant concentration as well as a simplified expression for the total fluorescence. These simplifications are possible because the varying functions (Eq. (21) and Eq. (24) respectively) are nearly constant. The proof of this fact can be found in Appendix B. Finally the equation for the spatial average, as in Eq. (30), involves these functions and it is shown that replacing them by constants creates a small error. This is shown in Appendix C.

## 6. Conclusion

The dynamics of the populations of excited and unexcited fluorophores are described by a pair of first order partial differential equations that incorporate the kinetics of the fluorophore reaction driven by a periodically varying laser beam and the convective fluid flow in a measurement apparatus. We obtain a dramatic reduction in the mathematical complexity of the model by taking advantage of the disparate time scales for excitation, relaxation and degradation. This is followed by averaging the reactant concentration over the laser beam, which is assumed to have a Gaussian profile. A single ordinary differential equation, Eq. (30), is obtained describing the evolution of the spatially averaged reactant concentration. The measured fluorescent signal, Eq. (19), is expressed in terms of the spatially averaged reactant concentrations. These equations provide a prediction of the response whenever the photochemical reaction degrades the fluorophores flowing past the laser beam.

Mathematical justification for the approximations used in the course of the derivation is given and some comparisons to the time independent case are described. The predicted frequency response (based on the time varying solution of Eq. (30)) is used to derive a functional form for the ratio of the in-phase and quadrature parts of the fluorescent signal. This same form provides a good fit for experimental data. The fit and the connection with physical parameters of the experiment will be discussed in a forthcoming paper.

## 7. APPENDIX A

This appendix addresses the issue of the validity of the perturbation expansion of *N*_0_ and *N*_1_ for large times as stated in Eq. (6) and Eq. (7). The discussion is based on the standard observation that the solutions of Eq. (6) and Eq. (7) can be expressed in terms of the solution of a parameterized system of ordinary differential equations. Here a new time variable *s* where *t* = *s* + *θ* is introduced. The relaxation rate is denoted by *k_r x_*.

εdN0ds(θ+s,y,ε)=−k¯a(x,y,θ+s)N0(θ+s,y,ε)+k¯rxN1(θ+s,y,ε)εdN1ds(θ+s,y,ε)=k¯a(x,y,θ+s)N0(θ+s,y,ε)−k¯rxN1(θ+s,y,ε)−εkdN1(θ+s,y,ε)dxds(θ+s,ε)=vN0(y,θ,ε)=N0,N1(y,θ,ε)=0,x(θ,ε)=−L2. (A1)

See for example the book Aris and Amundson [9], which contains many worked examples from the chemistry literature as well as a general discussion of the transformation of partial differential equations such as Eq. (6). This is known as the “method of characteristics.” The variable *θ* ≥ 0 parameterizes the family of solution curves that satisfy the differential equations in *s* and the initial conditions at *s* = 0 displayed on the last line. The original time variable is *t* = *θ* + *s* and this leads to the interpretation of *θ* as the time a particular family of particles entered the apparatus (at x=−L2) while *s* is the amount of time elapsed since entry. If we are following the set of particles that entered the apparatus at the beginning of the observation period, i.e., at *θ* = 0, then *s* = *t*. Here we will assume that θ≥−Lv*θ*. For each *θ*, a perturbation method will be used to construct solutions of Eq. (A1) based on the representation,

$$\begin{array}{c} N_{0}(y,\theta +s,\varepsilon )={\hat{N}}_{0}(y,\theta ,s,\varepsilon )+\eta_{0}(y,\theta ,\tau ,\varepsilon )\\ N_{1}(y,\theta +s,\varepsilon )={\hat{N}}_{1}(y,\theta ,s,\varepsilon )+\eta_{1}(y,\theta ,\tau ,\varepsilon )\\ x(\theta +s,\varepsilon )=\hat{X}(\theta ,s,\varepsilon )+\varepsilon \xi (\theta ,\tau ,\varepsilon ) \end{array}$$ (A2)

where ${\hat{N}}_{i}$ *i* = 0,1 refers to the large time component of ${\hat{N}}_{i}$ and *η_i_* is the small time or inner correction of the large time solution. A similar decomposition holds for *x*. The inner correction governs the behavior of the solution of A1 near the time of entry *s* = 0 and since we expect a rapid initial transient it is written in terms of a fast time scale τ=sε. The large time components have perturbation expansions

$$\begin{array}{c} {\hat{N}}_{0}(y,\theta ,s,\varepsilon )={\hat{N}}_{00}(y,\theta ,s)+\varepsilon {\hat{N}}_{10}(y,\theta ,s)+O(\varepsilon^{2})\\ {\hat{N}}_{1}(y,\theta ,s,\varepsilon )={\hat{N}}_{10}(y,\theta ,s)+\varepsilon {\hat{N}}_{11}(y,\theta ,s)+O(\varepsilon^{2})\\ \hat{X}(\theta ,s,\varepsilon )=X_{0}(\theta ,s)+\varepsilon X_{1}(\theta ,s)+O(\varepsilon^{2}). \end{array}$$ (A3)

If we replace *N*_0_, *N*_1_, *x* by their respective large time components in Eq. (A1) then,

$$\begin{array}{l} \varepsilon \frac{d{\hat{N}}_{0}}{ds}=-{\bar{k}}_{a}(\hat{X},y,\theta +s){\hat{N}}_{0}+{\bar{k}}_{rx}{\hat{N}}_{1}\\ \varepsilon \frac{d{\hat{N}}_{1}}{ds}={\bar{k}}_{a}(\hat{X},y,\theta +s){\hat{N}}_{0}-{\bar{k}}_{rx}{\hat{N}}_{1}-\varepsilon k_{d}{\hat{N}}_{1}\\ \frac{d\hat{X}}{ds}=v. \end{array}$$ (A4)

On setting *ε* = 0, one gets,

$$\begin{array}{l} {\hat{N}}_{10}(y,\theta ,s)=\frac{{\bar{k}}_{a}({\hat{X}}_{0}(\theta ,s),y,\theta +s)}{{\bar{k}}_{rx}}{\hat{N}}_{00}(y,\theta ,s)\\ \frac{dX_{0}}{ds}(\theta ,s)=v \end{array}$$ (A5)

the analogue of the quasi-equilibrium condition expressed in Eq. (9). Similarly when coefficients of *ε* on the left hand side of A4 are equated to those on the right, there results

$$\begin{array}{l} \frac{d{\hat{N}}_{00}}{ds}={\bar{k}}_{rx}{\hat{N}}_{11}-{\bar{k}}_{a}({\hat{X}}_{0}(s),y,\theta +s){\hat{N}}_{01}\\ -\left(\frac{\partial {\bar{k}}_{a}({\hat{X}}_{0}(s),y,\theta +s)}{\partial \hat{X}}{\hat{X}}_{1}\right){\hat{N}}_{00}\\ \frac{d{\hat{N}}_{10}}{ds}={\bar{k}}_{a}({\hat{X}}_{0}(s),y,\theta +s)){\hat{N}}_{01}\\ -{\bar{k}}_{rx}{\hat{N}}_{11}-{\bar{k}}_{d}{\hat{N}}_{10}+\left(\frac{\partial {\bar{k}}_{a}({\hat{X}}_{0}(s),y,\theta +s)}{\partial \hat{X}}{\hat{X}}_{1}\right){\hat{N}}_{00}\\ \frac{dX_{1}}{ds}=0. \end{array}$$ (A6)

Here we suppressed some of the arguments for ease of reading. Comparing this with Eq. (11) we see that for suitable initial conditions on *X_0_*, ${\hat{N}}_{00}$, and ${\hat{N}}_{10}$, $N(y,\theta ,s)={\hat{N}}_{00}(y,\theta ,s)+{\hat{N}}_{10}(y,\theta ,s))$ is a solution of Eq. (13) expressed in terms of the solution of the parameterized ordinary differential equation.

$$\begin{array}{c} \frac{dN(y,\theta ,s)}{ds}+\frac{k_{d}{\bar{k}}_{a}(X_{0}(s),y,\theta +s)}{{\bar{k}}_{rx}{\bar{k}}_{a}(X_{0}(s),y,\theta +s)}N(y,\theta +s)=0\\ \frac{dX_{0}(s)}{ds}=v. \end{array}$$

The appropriate initial conditions can be obtained by examining the behavior of the inner corrections. Equations for them are obtained by returning to A1 and replacing the functions *N*_0_, *N*_1_, and *x* and by the expressions on the right hand side of A2. We seek representations of the corrections in terms of the following perturbation expansion:

$$\begin{array}{l} \eta_{0}(\tau ,\varepsilon ,\theta )=\eta_{00}(\tau ,\theta )+\varepsilon \eta_{01}(\tau ,\theta )+O(\varepsilon^{2})\\ \eta_{1}(\tau ,\varepsilon ,\theta )=\eta_{10}(\tau ,\theta )+\varepsilon \eta_{11}(\tau ,\theta )+O(\varepsilon^{2})\\ \xi (\tau ,\varepsilon ,\theta )=\xi_{0}(\tau ,\theta )+\varepsilon \xi_{1}(\tau ,\theta )+O(\varepsilon^{2}). \end{array}$$ (A7)

We will suppress the dependence on *y* in what follows. After performing the previously described replacement involving A1and A2, and then using the right hand sides of A4, the desired equations are obtained. In them, the fast time variable is introduced by the change of variable, *s* = *ετ*. We will write down the resulting equations just for *η*_1_ and *ξ*,

$$\begin{array}{l} \frac{d\eta_{1}}{d\tau }={\bar{k}}_{a}(\hat{X}(\varepsilon \tau )+\varepsilon \xi (\tau ),\theta +\varepsilon \tau )[N_{0}(\varepsilon \tau )+\eta_{0}(\tau )]\\ -{\bar{k}}_{a}(\hat{X}(\varepsilon \tau )+\varepsilon \xi (\tau ),\theta +\varepsilon \tau )N_{0}(\varepsilon \tau )\\ -{\bar{k}}_{rx}[N_{1}(\varepsilon \tau )+\eta_{1}(\tau )]+k_{rx}N_{1}(\varepsilon \tau )\\ -\varepsilon {\bar{k}}_{d}[N_{1}(\varepsilon \tau )+\eta_{1}(\tau )]+\varepsilon k_{d}[N_{1}(\varepsilon \tau )+\eta_{1}(\tau )]\\ \frac{d\xi }{d\tau }=0. \end{array}$$ (A8)

The equation for *η*_0_ is quite similar to the one for *η*_1_.

The initial conditions of the original problem A1 can be used as the initial conditions for *η*_0_, *η*_1_, and *ξ* since these functions approximate the initial part of the solution of A1. Thus *η*_0_ (0) = *N*^0^, *η*_0_ (0) = 0, ξ(0)=−L2. Equations for the leading terms in the perturbation expansions in (A7) are then obtained by setting *ε* = 0 in (A8). There results,

$$\begin{array}{l} \frac{d\eta_{00}}{d\tau }=-{\bar{k}}_{a}(X_{0}(0),\theta )\eta_{00}(\tau )+{\bar{k}}_{rx}\eta_{10}(\tau )\\ \frac{d\eta_{10}}{d\tau }={\bar{k}}_{a}(X_{0}(0),\theta )\eta_{00}(\tau )-{\bar{k}}_{rx}\eta_{10}(\tau )\\ \frac{d\xi_{0}}{d\tau }=0. \end{array}$$ (A9)

The initial value *X*_0_(0) is a constant that will be determined shortly. Adding the first two equations in (A9) then gives us $\frac{d}{d\tau }\left(\eta_{00}+\eta_{10}\right)=0$ so from the just stated initial conditions it follows that *η*_00_(*τ*) +*η*10(*τ*) = *η*_00_ (0) + *η* (0) = *N*^0^− that is, the sum of the leading terms in the expansion of the inner correction of *N*_0_ + *N*_1_ is a constant. The next step in determining the initial conditions for the large time solution (which in general will not be the initial conditions for the original problem) is to match the large time or outer solution with the inner correction in an overlapping region of the *s* axis. This region is defined as the outer reaches of the initial interval where the inner correction is valid and the beginning of the region of validity of the large time solution. To do this we calculate the limit of the inner correction as *τ* → ∞ by fixing *s* and letting *ε* → 0. The limit must match the limit of the large time solution as *s* = *ετ* → 0 where *τ* is fixed and *ε* → 0. For our problem this is written as,

N0=limτ→∞(η00(τ)+η10(τ))=lims→0(N00(s)+N10(s))−L2=limτ→∞ξ0(τ)=lims→0X0(s). (A10)

In this way, we determine the initial condition for the leading order large time solution *N*(*y*, *θ*, *s*) and thereby determine the boundary condition. Indeed from the second equation in A10, the initial condition for the *x* position to leading order is X0(0)=−L2. Given the large time solution and inner corrections formally constructed here, we can for each *θ* ≥ 0 construct a solution of A1 that is uniformly valid throughout the interval 0 ≤ s ≤ *T*/*ε* for each fixed *ε* > 0 Standard theorems in the theory of singular perturbations show that the operations used here to obtain it are rigorously justified. The key hypothesis that must be satisfied in our problem is that the inner solution must approach a finite limit exponentially. Recall that *η*_10_ = *N*^0^ − *η*_00_. Substituting this relation into the first equation in A9 results in the following equation for *η*_00_.

$$\begin{array}{c} \frac{d\eta_{00}}{d\tau }+\left[{\bar{k}}_{a}\left(X_{0}\left(0\right),\theta \right)+{\bar{k}}_{rx}\right]\eta_{00}={\bar{k}}_{rx}N^{0}\\ \eta_{00}\left(0\right)=N^{0}. \end{array}$$ (A11)

Solving this constant coefficient equation shows that *η*_00_ and thus *η*_10_ has the required behavior as *τ* → ∞. Note that equation (A1) is not a standard singular perturbation problem because the Jacobian associated with the reduced problem, i.e., the problem obtained from setting *ε* = 0 in the first two equations of (A1), is singular. Validity of the singular perturbation method in this “critical” case was proved by Vasil’eva and Butuzov [10] and was treated analytically and numerically by O’Malley and Flaherty [11]. These results require that the Jacobian have a constant deficient rank *k* for all *s* and its non-zero eigenvalues have negative real parts. A discussion of these problems can be found in O’Malley [12] and Smith [13].

A complete solution of (A1) uniformly valid in an interval comes from combining the large time and inner correction and then subtracting the common value they share in the overlapping region. If *N*(*y*, *θ*, *s*) is the total fluorophore population to leading order we have,

N0(y,θ+s,ε)=k¯rxN(y,θ,s)k¯a(X^0(θ,s),y,θ+s)+k¯rx+k¯rx(1−e−(k¯¯a+krx)sε)k¯¯a+k¯rxN0+N0e−(k¯¯a+krx)sε−k¯rxN0k¯¯a+k¯rx+O(ε) (A12)

where k¯¯a=k¯a(X0(0),y,θ)=k¯a(−L2,y,θ). Note that when *s* is large

$$N_{0}\left(y,\theta +s,\varepsilon \right)=\frac{{\bar{k}}_{rx}N\left(y,s,\theta \right)}{{\bar{k}}_{a}\left(X_{0}\left(s\right),y,\theta +s\right)+{\bar{k}}_{rx}}+O\left(\varepsilon \right)+\delta \left(s\right).$$ (A13)

Similar reasoning shows that

$$\begin{array}{c} N_{1}\left(y,\theta +s,\varepsilon \right)=\frac{{\bar{k}}_{a}\left(X_{0}\left(s\right),y,\theta +s\right)N\left(y,\theta ,s\right)}{{\bar{k}}_{rx}+{\bar{k}}_{a}\left(X_{0}\left(s\right),y,\theta +s\right)}\\ +O\left(\varepsilon \right)+\beta \left(s\right) \end{array}$$ (A14)

where *δ* (s) and *β* (s) are exponentially small in *s* and *O*(*ε*) holds uniformly in the interval 0≤s≤Tε and where X0(s)=−L2+vs. On adding Eqs. (A13) and (A14) we see that at large times, the total fluorophore population is dominated by *N*(*y*, *θ*, *s*). It is in this sense that we can justify the statements in Sec. 2.

## 8. APPENDIX B

We will write *N*(*r, t*) = Φ(*r*) + Ψ(*r*, *t*) where $\Phi \left(r\right)=\underset{T\to \infty }{\lim}\frac{1}{T}\int_{0}^{T}N\left(r,t\right)dt$ and Ψ(*r*, *t*) = *N*(*r*, *t*) − Φ(*r*). We begin by showing that Ψ(*r*, *t*) and ∂Ψ(r,t)∂x are small when the parameter *η* is small. To obtain an expression for Ψ we will first derive an equation for Φ from Eq. (20), by applying the operator $\underset{T\to \infty }{\lim}\frac{1}{T}\int_{0}^{T}\left\{\right\}$.

One finds Φ that must satisfy

v∂Φ(r)∂x=−ηPof(r)1+bP0f(r)Φ(r)−L2≤x≤L2,−a≤y≤aΦ(r)|x=−L2=N0. (B1)

We assume that all of the limiting averaging operations used to derive B1 are valid. This will be the case for example when the integrands are quasi-periodic [14] – a reasonable assumption for this problem. The boundary condition at x=−L2, comes from applying the operator $\underset{T\to \infty }{\lim}\frac{1}{T}\int_{0}^{T}\left\{\right\}$ to the boundary condition Eq. (15). Substituting Eq. (B1) into Eq. (20) will lead to an equation for Ψ.

∂Ψ(r,t)∂t+v∂Ψ(r,t)∂x+H(r,t)Ψ(r,t)=g(r,t)H(r,t)=ηP(r,t)1+bP(r,t),g(r,t)=(−H(r,t)+ηP0f(r)1+bP0f(r))Φ(r)Ψ(r,t)|x=−L2=0,Ψ(r,0)=−Φ(r),x>−L2. (B2)

The conditions for Ψ at x=−L2, are obtained from the conditions for Φ and *N*. Note the discontinuity in the value of Ψ at *t* = 0 as particles are pumped into the vessel. It propagates to larger values of *x* for *t* > 0 as a shock. However since here we will assume that t>(x+L2)v for any *x* (for example if t>Lv the shock will have passed and we have continuity in Ψ. The solution of Eq. (B2) for t>(x+L2)v is

$$\Psi \left(r,t\right)=\frac{1}{v}\int_{\frac{-L}{2}}^{x}\exp\left\{-\frac{1}{v}\int_{x^{\prime }}^{x}H\left(\xi ,y,\tau \right)d\xi \right\}g\left(x^{\prime },y,\tau \right)dx^{\prime }$$ (B3)

where $\tau =\left(x,\xi ,t\right)=t-\left(\frac{x-\xi }{v}\right)$. The derivative with respect to *x* is written,

$$\begin{array}{l} v\frac{\partial \Psi \left(r,t\right)}{\partial x}=\\ -\frac{H\left(r,t\right)}{v}\Psi +\frac{1}{V}\int_{\frac{-L}{2}}^{x}\left(\int_{x^{\prime }}^{x}H_{\tau }d\xi \right)\exp\left\{-\frac{1}{V}\int_{x^{\prime }}^{x}Hd\xi \right\}gdx^{\prime }\\ +g\left(r,t\right)-\;\frac{1}{V}\int_{\frac{-L}{2}}^{x}\exp\left\{-\frac{1}{v}\int_{x^{\prime }}^{x}Hd\xi \right\}g_{\tau }dx^{\prime } \end{array}$$ (B4)

where for easier reading we have suppressed some of the function arguments. The functions *H_t_* and *g t* are derivatives

$$\begin{array}{cc} H_{\tau }=\frac{\partial H\left(\xi ,y,\tau \right)}{\partial \tau }, & g_{\tau } \end{array}=\frac{\partial g\left(x^{\prime },y,\tau \right)}{\partial \tau }.$$ (B5)

From Eq. (B2) it can be seen that *g_t_* = − *H_t_* |_*ξ* = *x′*_ Φ(*r*). To show that | Ψ(*r*, *t*) | and $\left|\frac{\partial \Psi \left(r,t\right)}{\partial x}\right|$ are small we will show that *g*, *H*, *H_τ_* are all small. Indeed from Eq. (B1) we have Φ(*r*) ≤ *N*^0^ so | *g_t_* | will be small if | *H_τ_* | is. Using the fact that *P*(*r, t*) = (*P*_0_ + Δ(*t*)) *f* (*r*) where Δ(*t*) = *P*_1_cos(*ωt*) we find that

$$\frac{\partial H\left(r,\tau \right)}{\partial \tau }=\frac{\eta f\left(r\right)\omega P_{1}\sin\left(\omega \tau \right)}{{\left[1+bP\left(r,t\right)f\left(r\right)\right]}^{2}}.$$ (B6)

We can perform a similar analysis of the remaining terms in Eq. (B3) and Eq. (B4). Using the notation *O*(*ρ*) to denote a term whose absolute value remains bounded by a multiple of *ρ* as *ρ →* 0, the results are summarized

$$\begin{array}{l} \frac{\partial H(r,\tau )}{\partial \tau }=O(\eta )\\ \frac{\partial g(r,\tau )}{\partial \tau }=O(\eta )\\ H(r,\tau )=O(\eta )\\ g(r,\tau )=\frac{\eta \Delta (t)f(r)}{\left(1+bP_{0}f(r))(1+bP(r,t)f(r)\right)}=O(\eta ) \end{array}$$ (B7)

These relations hold uniformly for all *t* ≥ 0 since all the time varying functions in (B7) are periodic. Thus by considering *η* small enough we can conclude that for all *t* ≥ 0

$$\begin{array}{l} \Psi (r,t)=O(\eta ),\;\frac{\partial \Psi (r,t)}{\partial x}=O(\eta )\\ N(r,t)=\Phi (r)+O(\eta ),\;\frac{\partial N(r,t)}{\partial x}=\frac{\partial \Phi (r)}{\partial x}+O(\eta ). \end{array}$$ (B8)

Here we used the relation *N*(*r, t*) = Φ(*r*) + Ψ(*r, t*).

Finally we turn to a discussion of the replacement of the functions $\bar{\alpha }(t)$ and ${\bar{\alpha }}^{\prime }(t)$ in Eq. (22), Eq. (24), and Eq. (25) by constants *α* and *α*′. We use the fact that the constants are

$$\alpha =\frac{\iint_{R}f(r)\Phi (r)f(r)dr}{\iint_{R}f(r)\Phi (r)dr},\alpha^{\prime }=\frac{\iint_{R}f(r)\frac{\partial \Phi (r)}{\partial x}f(r)dr}{\iint_{R}f(r)\frac{\partial \Phi (r)}{\partial x}dr}$$ (B9)

to write $\bar{\alpha }(t)=\alpha +a(t)$ where

$$a(t)=\frac{\left(\iint_{R}f\Phi \right)\left(\iint_{R}f\Psi f\right)\left(\iint_{R}f\Phi f\right)\left(\iint_{R}f\Psi \right)}{\left(\iint_{R}f[\Phi +\Psi ]\right)\left(\iint_{R}f\Phi \right)}.$$ (B10)

Applying Eq. (B8) for Ψ to the numerator of (B10) we see that

$$\bar{\alpha }(t)=\alpha +O(\eta ).$$ (B11)

In an analogous fashion the function ${\bar{\alpha }}^{\prime }(t)={\bar{\alpha }}^{\prime }+\alpha^{\prime }(t)$ where

$$a^{\prime }(t)=\frac{\left(\iint_{R}f\frac{\partial \Phi }{\partial x}\right)\left(\iint_{R}f\frac{\partial \Psi }{\partial x}f\right)-\left(\iint_{R}f\frac{\partial \Phi }{\partial x}f\right)\left(\iint_{R}f\frac{\partial \Psi }{\partial x}\right)}{\left(\iint_{R}f[\frac{\partial \Phi }{\partial x}+\frac{\partial \Psi }{\partial x}]\right)\left(\iint_{R}f\frac{\partial \Phi }{\partial x}\right)}.$$ (B12)

Using Eq. (B8) for $\frac{\partial \Psi }{\partial x}$ and applying it to Eq. (B12) therefore implies the equation

$${\bar{\alpha }}^{\prime }(t)=\alpha^{\prime }+O(\eta ).$$ (B13)

Equations (B11) and (B13) hold uniformly for all *t* ≥ 0. Thus the effect of replacing $\bar{\alpha }(t)$ by the constant *α* is to produce an error of size *O*(*η*) by Eq. (B11) and Eq. (B13). This argument also justifies the replacement in Eq. (19).

## 9. APPENDIX C

In Sec. 3 an equation Eq. (26) for the evolution of the spatially averaged reactant concentration is derived from Eq. (20). To properly assess the effect of replacing the functions $\bar{\alpha }(t)$ and ${\bar{\alpha }}^{\prime }(t)$ respectively with constants *α* and *α*′ in the derivation of Eq. (26) it must first be recognized that the time derivative of *N*(*r, t*) is small since it is *O*(*η*). This can be seen from the definition of Ψ in Appendix B and Eqs. (B2), (B7), and (B8). We must therefore show that the neglected terms are of smaller asymptotic order. Before the replacement, the left hand side of Eq. (20) after multiplication by *f* (*x, y*) and integration is actually Eq. (22). Using the notations of Appendix B, Eq. (22) is rewritten,

$$\begin{array}{l} \frac{\partial \langle N(t)\rangle }{\partial t}+b(P_{0}+\Delta (t))\frac{\partial }{\partial t}\left\{(\alpha +a(t))\iint_{R}f(r)N(r,t)dr\right\}\\ =[1+b(P_{0}+\Delta (t))\alpha ]\frac{\partial \langle N(t)\rangle }{\partial t}\\ +b(P_{0}+\Delta (t))\left\{\left(\frac{\partial a(t)}{\partial t}\right)\langle N(t)\rangle +a(t)\frac{\partial \langle N(t)\rangle }{\partial t}\right\}. \end{array}$$ (C1)

The term $a(t)\left(\frac{\partial \langle N(t)\rangle }{\partial t}\right)$ is *O*(*η*^2^) thanks to Eqs. (B8) and (B11) and can therefore be neglected. The term $b(P_{0}+\Delta (t))\left(\frac{\partial a(t)}{\partial t}\right)\langle N(t)\rangle$ is proportional to *ηbP*_1_(see Eq. (B5), Eq. (B6), and Eq. (B10)). Thus this term can be dropped when either *P*_1_ or *b* is small. Note that if the constant *α* is large, (i.e., *α* >> 1) the following terms are retained because they are going to be of larger order than the terms that are dropped.

$$\begin{array}{l} b(P_{0}+\Delta (t))\alpha \left(\frac{\partial \langle N(t)\rangle }{\partial t}\right)=O(\eta bP_{1}\alpha )\\ -\eta \alpha (P_{0}+\Delta (t))\langle N(t)\rangle =O(\eta \alpha ) \end{array}$$

The first expression comes from the left hand side of C1 and it is retained, and the second expression comes from the right hand side of Eq. (20) after multiplication and integration, i.e., Eq. (25). Therefore let us write the right hand side of Eq. (20) after multiplication by *^f^*(*x, y*) and integration as,

$$\begin{array}{l} -v(1+b\left(P_{0}+\Delta \left(t\right)\right)\left(\alpha^{\prime }+a^{\prime }\left(t\right)\right)\alpha \int \int_{R}f\left(r\right)\frac{\partial N\left(r,t\right)}{\partial x}dr\\ -\eta \left(\alpha +a\left(t\right)\right)\left(P_{0}+\Delta \left(t\right)\right)\langle N\left(t\right)\rangle . \end{array}$$ (C2)

We observe that

$$\begin{array}{l} -\eta a\left(t\right)\left(P_{0}+\Delta \left(t\right)\right)\langle N\left(t\right)\rangle =Ο\left(\eta^{2}\right)\\ -vb\left(P_{0}+\Delta \left(t\right)\right)a^{\prime }\left(t\right)\int \int_{R}f\left(r\right)\frac{\partial N\left(r,t\right)}{\partial x}dr=Ο\left(\eta b\right) \end{array}$$

because of Eq. (B11) and Eq. (B13). Again taking *b* small means that both these terms can be dropped. Moreover if *α*′ ≫ 1, the term $b\left(P_{0}+\Delta \left(t\right)\right)\alpha^{\prime }\int \int_{R}f\left(r\right)\frac{\partial N\left(r,t\right)}{\partial x}dr$ is retained. Thus, the remaining terms in Eq. (C1) form the left hand side of Eq. (26) while the remaining terms in Eq. (C2) become the right hand side of Eq. (26).
